# Cardiac damage in autoimmune diseases: Target organ involvement that cannot be ignored

**DOI:** 10.3389/fimmu.2022.1056400

**Published:** 2022-11-22

**Authors:** Shu-Yue Pan, Hui-Min Tian, Yong Zhu, Wei-Jie Gu, Hao Zou, Xu-Qiang Wu, Rui-Juan Cheng, Zhi Yang

**Affiliations:** ^1^ Department of Rheumatology and Immunology, The Fifth People’s Hospital Affiliated to Chengdu University of Traditional Chinese Medicine/Chengdu Fifth People’s Hospital, Chengdu, China; ^2^ Department of Rheumatology and Immunology, West China Hospital, Sichuan University, Chengdu, Sichuan, China; ^3^ Department of Radiology, The Fifth People’s Hospital Affiliated to Chengdu University of Traditional Chinese Medicine/Chengdu Fifth People’s Hospital, Chengdu, China

**Keywords:** autoimmune disease, cardiac damage, rheumatoid arthritis, systemic lupus erythematosus, polymyositis/dermatomyositis, Sjogren’s Syndrome, systemic sclerosis

## Abstract

Autoimmune diseases are diseases that cause damage to the body’s own tissues as a result of immune dysfunction, often involving multiple organs and systems. The heart is one of the common target organs of autoimmune diseases. The whole structure of the heart can be affected, causing microcirculatory disorders, arrhythmias, pericardial damage, myocarditis, myocardial fibrosis, and impaired valvular function. However, early clinical manifestations of autoimmune heart damage are often overlooked because they are insidious or have no typical features. The damage is often severe and irreversible when symptoms are apparent, even life-threatening. Therefore, early detection and treatment of heart damage in autoimmune diseases is particularly important. Herein, we review the clinical features and mechanisms of cardiac damage in common rheumatic diseases.

## Introduction

Autoimmune diseases are disorders of the immune system that cause damage to body’s own tissues and organs, leading to disability and death. The pathogenesis of autoimmune diseases is complex and often involves multiple organs and systems of the body (1).

The clinical manifestations of cardiac damage in autoimmune diseases are complex. The mechanism of cardiac damage is not clear and considered to be related to a combination of autoimmune regulatory disorders and chronic inflammation. Immune complex deposition in different areas of the heart can cause autoimmune inflammatory reactions and activate body’s complement system (2). Oxidative stress caused by persistent inflammation, along with cytokine-induced increased fibroblast activity, can lead to myocardial collagen deposition and myocardial interstitial fibrosis (3). In addition, chronic inflammation leads to accumulation of monocyte in the blood and promotes upregulation of intercellular adhesion molecules, the release of pro-inflammatory cytokines, and the production of matrix degrading enzymes. This will lead to vascular endothelial dysfunction and early onset of coronary atherosclerosis (4). The mechanism and extent of cardiac involvement vary across autoimmune diseases, which results in varied pathophysiology and clinical manifestations. The aim of this review is to summarize clinical manifestations and mechanism of cardiac damage in systemic autoimmune diseases (**Table 1**).

**Table 1:** Common cardiac damage in autoimmune diseases.

Disease	Common Heart Damage
**RA**	**Heart failure:** left ventricular diastolic dysfunction. (5–11) **Coronary artery injury:** including coronary arteritis, coronary thrombosis and atherosclerotic coronary disease. (12, 13) **Pericarditi**s: simple fibrinous pericardial obstruction and chronic pericardial effusion. (14–18) **Arrhythmia:** ST-T wave change, QTc prolongation, heart block, atrial fibrillation. (19–22) **Heart valve involvement:** nonspecific fibrosis and valve thickening, mainly involves the mitral and aortic valves. (23, 24)
**SLE**	**Pericardial lesions:** pericardial thickening and pericardial effusion, pericarditis. (25–29) **Myocarditis:** accompanied by myocardial fibrosis and scar formation. (30–32) **Heart valve disease:** mainly valve thickening, excrescence, regurgitation and stenosis. (33–38) **Arrhythmia:** sinus tachycardia, supraventricular arrhythmias, atrioventricular block (especially high grade), various extrasystoles, sick sinus syndrome, atrial fibrillation. (39–43)
**PM/DM**	**Myocarditis:** myocardial fibrosis (swollen, showing degeneration and necrosis). (44–47) **Arrhythmias:** premature atrial or ventricular contractions, atrial or ventricular tachycardia, atrial fibrillation. (47, 48) **Conduction abnormalities:** Bundle branch block and atrioventricular block were predominant in conduction block, with left anterior fascicular block and right bundle branch block being the most common. (48, 49)
**pSS**	**Myocardial injury:** myocarditis, myocardial fibrosis and myocardial ischemia. (50–53) **Cardiac valve changes:** valvular regurgitation (mitral, tricuspid and aortic valves). (54)
**SSc**	**Myocardial fibrosis:** often located under the endocardium, resulting in reduced systolic function and compliance. (55–58) **Coronary artery spasm and stenosis**. (55–58)

## Rheumatoid arthritis

Rheumatoid arthritis (RA) is the most common chronic systemic autoimmune disease. The pathological changes of RA include chronic inflammation of the synovial membrane, pannus formation and gradual destruction of articular cartilage and bone. These processes ultimately lead to joint deformity and loss of function (59). Early cardiac diseases are very common but often subclinical in patients with RA. However severe cardiac damage increases the risk of ischaemic heart disease and heart failure and is a leading cause of premature death in patients (5, 6). Most RA patients do not develop severe cardiac manifestations even after years of disease: their hearts predominantly exhibit subclinical and asymptomatic changes. RA cardiovascular injuries mainly include heart failure, ischemic heart disease, pericarditis, myocarditis, cardiomyopathy, cardiac amyloidosis, coronary vasculitis, arrhythmias and valvular diseases (7). The increased risk of sudden cardiac death in RA patients is secondary to ischemic heart disease, non-ischemic heart disease and cardiac arrhythmias (60). At the coronary level, endothelial dysfunction in large and small coronary arteries can lead to ischemic heart disease, either secondary or not secondary to atherosclerosis. At the myocardial level, the presence of local inflammation (myocarditis, pericarditis) and fibrosis may cause diastolic or systolic dysfunction and subsequent cardiac hypertrophy. These changes in the coronary vasculature and myocardial tissue can lead to cardiac arrhythmias, hear failure and sudden cardiac death (61).

Heart failure is one of the main causes of cardiovascular mortalities in patients with RA (8). Studies have shown that RA patients have a higher incidence of heart failure with preserved ejection fraction (HFpEF) compared to non-RA patients (9). Patients with RA also have an increased incidence of congestive heart failure (CHF), and inflammatory stimuli appear to play an important role in the development of CHF in patients with RA (10). Left ventricular diastolic dysfunction is a common manifestation of RA heart failure, the risk of which increases as the disease progresses (11) RA induced heart failure do not typically present with systolic dysfunction. Many symptom reliefing drugs for RA, such as steroid and NSAIDs, carry a potential risk of heart damage (12). RA predisposes patients to a variety of coronary artery injury, such as coronary arteritis, vasospasm and microvascular disease. All of these can destabilize atherosclerotic coronary artery disease and lead to cardiac ischemia (13). In addition, glucocorticoid use is associated with an increased risk of heart failure, myocardial infarction, stroke and all-cause mortality in a dose-dependent manner (12).

Pericarditis is another common cardiac complication in RA. Although pericarditis with clinical symptoms may be less than 15%, echocardiography can show 20%~50% of RA patients with pericardial involvement, of which 20%~40% are pericarditis, mainly fibrinous (14, 15). RA pericarditis generally has a favorable prognosis and rarely results in cardiac tamponade. However, fibrinous pericarditis can lead to pericardial constriction and even death (16). Pericarditis in RA is characterized pathologically by diffused fibrinoid exudation of the pericardium and varying degrees of adhesions to the heart. The main clinical manifestations are due to fibrinous pericardial occlusion or chronic pericardial effusion; a small number of patients may present with fever, chest pain, an enlarged heart border and pericardial friction rub in the acute phase. Their clinical presentation may not correlate with the duration or severity of their joint disease. Symptoms are often vague and nonspecific, which frequently cause delays in diagnosis and subsequent treatment (17). RA pericarditis is mainly caused by chronic inflammatory reactions, whereas pericardial effusion is mostly caused by extensive vasculitis, which can precede joint disease. Severe cases associated with cardiac tamponade, pericardial effusion causing hemodynamic compromise, or constrictive pericarditis may require surgical treatment (e.g. pericardiocentesis, pericardiectomy, or pericardiotomy) (18).

Cardiac arrhythmia is an important clinical manifestation of cardiac damage caused by RA. Inflammatory processes in the heart can cause excessive myocardial fibrosis leading to ventricular sclerosis, which result in systolic and diastolic dysfunction as well as cardiac arrhythmias. Atrial fibrillation is the most common cardiac arrythmias in RA related arrythmias. Dysfunction of the immune system leading to arrhythmia in patients with RA may have the following mechanisms: (1) crosstalk between immune cells and fibroblasts and/or cardiomyocytes, leading to fibrosis and thus atrial fibrillation; (2) immune cells directly participate in the electrical remodeling of leukocytes through gap junctions with CX-43 leading to atrial fibrillation; (3) the immune system can directly affect the specific ion channel function on the surface of cardiomyocytes through autoantibodies and/or inflammatory cytokines leading to atrial fibrillation (19–21). RA patients progressively developed proarrhythmic QTc prolongation over time (22). Dai et al, reported in a recent animal study that RA can induce atrial fibrillation by increasing the number of RA rat cardiac fibroblasts and expressing inflammatory factors such as TNF-α and IL-6. These inflammatory factors are involved in pathophysiological processes such as atrial fibrosis (AF), atrial myocardial apoptosis and autophagy, as well as atrial electrical remodeling and autonomic remodeling. It has been found that the duration of AF is positively correlated with plasma IL-6 and TNF-α levels (62).

In addition, valvular involvement in patients with RA is also common in clinical practice. There are two types of heart valve involvement: (1) nonspecific fibrosis and sclerosis and thickening of the valve. The lesions are mostly locate at the base of the leaflets and annulus. Antigen-antibody complexes are deposited at the lesion site so that collagen fibers undergo fibroid degeneration, interstitial edema and inflammatory cell infiltration, which can lead to severe valvular distortion, deformation and poor coaptation (23); (2) a relatively specific form of involvement, where nodules, similar to those formed in subcutaneous tssues, develop in the myocardium and valvular tissue. Rheumatic granulomas can invade the annulus and base of the valve, and the lesions are mostly located in the center of the leaflet thus do not affect the surrounding part. The main valves involved are mitral and aortic valves, with valvular fibrosis causing insufficiency being the most common (24). The signs and symptoms of patients with heart valve disease induced by RA are insidious and not easily detected by clinical examination. Therefore, it is necessary to use ultrasonography to determine whether the heart valve is damaged and whether lesions have occurred.

## Systemic lupus erythematosus

The heart is one of the target organs involved in systemic lupus erythematosus (SLE), and all parts of the heart can be affected, including the pericardium, myocardium, endocardium, conduction system and coronary artery. Some scholars believe that immune complexes formed by specific antibodies such as ds-DNA, anti-Sm antibody and self-antigens exist in any tissues of the heart in SLE patients. These antibodies induce lymphocytes, immune factors, etc., to accumulate widely around the myocardium, pericardium, coronary vessel wall and conduction system, further promoting the inflammatory cascade response and causing cardiac tissue damage, dysfunction and cardiac dysrhythmia (63). Systemic manifestations of SLE are severe, and cardiac damage caused by SLE is easily overlooked in the early stage due to inconspicuous symptoms or subtle clinical signs and is difficult to manage as the disease progresses (64).

The main manifestations of pericardial diseases in SLE are pericardial thickening and pericardial effusion. Patients present clinically with precordial pain, shortness of breath, chest tightness, transient pericardial friction, etc. Histologically, acute pericardial involvement is characterized by exudative or fibrinous changes, if injury persists and recurs, scarring progresses can be observed as well (25). Pericardial diseases often occur in the early or recurrent stages of SLE, suggesting that pericardial diseases, especially pericardial effusion, are related to SLE activity. The autoimmune reactions during SLE activity produce immune complex deposition in the pericardium, causing increased pericardial permeability and lead to pericardial effusion. Serous or fibrinous exudations are seen in the acute phase, and pericardial adhesions may occur in the chronic phase (26). Inflammatory exudates and autoantibodies dominated by neutrophils were found in pericardial effusions of SLE patients (27). In addition, pericarditis is also a common manifestation of pericardial damage in SLE, typical histopathology is characterized by monocyte infiltration, deposition of fibrin substances and immune complexes (28, 29).

SLE also has a high rate of myocardial injury. Frequently the cardiomyopathy that SLE causes are occult and atypical clinical features. Autopsy revealed SLE with myocardial damage in up to 63% of the patients (30). Myocarditis is a serious manifestation of SLE myocardial injury, and myocarditis has a variety of clinical presentations, including dyspnea, non-exertional chest pain, peripheral edema, fever, diaphoresis, paroxysmal nocturnal dyspnea, nausea, vomiting, and palpitations (31). Immune complexes abnormally deposited in the myocardium can cause edema of connective tissue, infiltration of inflammatory cells, and eventually leading to degeneration and necrosis of cardiomyocytes, myocardial fibrosis and scarring., Biopsies typically show fibrotic plaques in the myocardium, interstitial mononuclear cell infiltration, and occasional myocardial cell necrosis with immune complex deposition, which can be observed even in areas without inflammatory lesions (32).

SLE with valvular heart disease is also a common clinical manifestation of heart injury. Roldan et al. identified valvular abnormalities in 61% of SLE patients by echocardiography; valve thickening was 51%, vegetations 43%, valve regurgitation 25%, and stenosis 4% (33). Under the microscope, the valvular lesions of SLE are characterized by proliferating and degenerated cells forming fibrin and fibrous tissue, occasionally with thrombi and necrotic tissue. They are most commonly located in the supraspinous tendon, papillary muscle, and at the edge of the valve (34). It is usually characterized by valve thickening, varying degrees of fibrosis, scar formation, calcification and valve excrescence. Clinical manifestations may include fever, bradycardia, tachycardia and cardiac murmur (35). Anticardiolipin antibodies are generally considered to be closely related to SLE valvular involvement (36), mainly mitral and aortic valves, leading to valve thickening, fibrosis, stenosis and dysfunction. Shapiro et al. found that immunoglobulin and complement were selectively deposited along the vascular wall, indicating that circulating immune complexes may participate in the formation of uninfected vegetation on the valve (37). At present, it is speculated that SLE with valvular disease may be caused by valvular inflammation from the deposition of circulating immune complexes and the activation of complement. Valvulitis and scarring promote vascular thickening and deformation, leading to valve dysfunction in elderly SLE patients (38). In addition, some scholars believe that antiphospholipid antibodies may contribute to the occurrence of valvular heart disease and promote thrombosis, but further studies into this area is required to confirm this. Severe valvular disease leads to ventricular enlargement and CHF, this is linked with poor prognosis.

The mechanisms of cardiac arrhythmias with SLE are not fully understood. The manifestations of arrhythmia in SLE patients are complex and diverse. Sinus tachycardia has the highest incidence, which may be caused by abnormal autonomic regulation of the sinoatrial node due to cardiac immune damage, excessive sympathetic tone and vagal weakening. This was followed by sinus bradycardia, which may be related to sinoatrial node damage leading to reduced function. Studies have shown that supraventricular arrhythmias, atrioventricular block (especially high grade) and other arrythmias are mostly associated with positive ribonucleoprotein (RNP) antibodies (39). In addition, it has been reported that anti-SSA and SSB antibodies in the serum of mothers with SLE can be transplacentally transmitted to the fetus, causing congenital heart block in newborns (36). Another study by Logar et al. suggested that anti-SSA and/or anti-SSB antibodies are associated with the development of conduction block in adult SLE patients (40). Studies have shown that there is a significant correlation between left atrial enlargement and AF (41). Other arrhythmias seen in SLE patients also include atrioventricular block, bundle branch or branch block, various extrasystoles, sick sinus syndrome, atrial fibrillation, etc. (42, 43).

## Polymyositis/dermatomyositis

Polymyositis/dermatomyositis (PM/DM) is a heterogeneous group of diseases characterized by chronic inflammation of striated muscle and skin. PM/DM can involve multiple organs and a significant proportion of patients have cardiac damage which affect the treatment and prognosis. The two types of myositis have different histopathological features. DM is a microangiopathy with capillary injury and secondary ischemic changes in muscle fibers, whereas PM has cellular infiltration at major endomysial sites with CD8+ lymphocytes infiltration into MHC I positive muscle fibres (44). The main manifestations of cardiac injury are arrhythmia, myocardial injury, pericarditis, valvular disease, pulmonary arterial hypertension and myocardial ischemia. Clinically, patients may have elevation of myocardial injury markers (TnT, brain natriuretic peptide), various rhythmic abnormal ECG findings, and systolic and diastolic dysfunction detected by echocardiogram (45). Myocardial cells can also be affected in inflammatory myopathy. Infiltration of inflammatory cell in myocardium is the most common pathological change, accounting for approximately 38% of patients, followed by focal myocardial fibrosis (22%) (46). Myocardial damage caused by PM/DM was highly similar to the pathological characteristics of skeletal muscle, mainly manifested as diffuse mononuclear cell infiltration in the myocardial interstitium and perivascular area. Additionally, the myocardium of the patient may experience fibrosis, showing enlargement, degeneration, and necrosis. Cardiac damage, as a common complication of PM/DM, has complex and diverse manifestations, has an insidious onset and varying severity of symptoms, requiring high vigilance in clinical practice.

Myocarditis may be one of the most important manifestations for cardiovascular system involvement in PM/DM patients. The main effect of myocarditis including myocardial hypertrophy, myocardial ischemia, myocardial infarction, cardiac enlargement and left ventricular systolicand diastolic dysfunction. The abnormal immune system of DM/PM patients attacks their own skeletal muscle cells while also attacking cardiomyocytes, resulting in varying degrees of cardiomyocyte involvement. Myocardial biopsy in patients with DM shows CD4+ T lymphocyte infiltration in the surface layer of the muscle bundles and the epimysial region that surrounds small vessels. There is also tissue atrophy around muscle bundles. Similar changes can be found in the cardiac conduction system as well.

Serum muscle enzyme examination is an important basis for the diagnosis of PM/DM. In addition to elevated creatine kinase levels, there may be elevated levels of cardiac enzymes such as cardiac troponin I (47). Cardiac myocardial enzyme changes can be masked by serum muscle enzyme changes in extensive skeletal muscle damage. Additionally, the clinical symptoms of myocardial injury are insidious, atypical symptoms and are easily ignored.

Arrhythmia and conduction abnormalities are most common in PM/DM myocardial damage. Electrocardiogram and Holter abnormalities observed in PM/DM included premature atrial or ventricular contractions, atrial tachycardia, ventricular tachycardia, atrial fibrillation, atrioventricular block, bundle branch block, abnormal Q waves, and nonspecific ST-T wave changes (49). Bundle branch block and atrioventricular block were predominant in conduction block, with left anterior fascicular block and right bundle branch block being the most common (48). The cardiac conduction system is affected by immune processes, such as myocarditis and myocardial fibrosis (involving the sinoatrial node and conduction system), accompanied by lymphocyte infiltration and contraction band necrosis. In addition, cardiac small vessel disease, such as luminal narrowing, smooth muscle hyperplasia and vascular intimal hyperplasia, can also cause arrhythmia, cardiac strangulation and other symptoms. The prognosis of PM/DM is related to the degree of cardiac involvement, so the detection and treatment of early cardiac dysfunction is critical and can prevent serious complications.

## Primary Sjogren’s syndrome

Primary Sjogren’s syndrome (pSS) is a diffuse connective tissue disease characterized by invasion of exocrine glands (such as salivary glands and lacrimal glands), abnormal proliferation of B lymphocytes and histolymphatic invasion. Heart is one the the target organis and pathologies are often subclinical and easily ignored. When pSS involves the heart, it mainly manifests as pericardial effusion, myocardial ischemia, heart block, valvular heart disease, etc., and even causes heart failure and myocardial infarction in severe cases. Cardiac involvement may be caused by myocarditis, myocardial fibrosis, vasculitis, or microvascular dysfunction, which is associated with chronic inflammatory responses resulting from autoimmune diseases, such as the effects from inflammatory agents like IL-6 and INF-α. Although the clinical manifestations of cardiac damage in patients with pSS are not obvious, multiple studies have shown that cardiac damage caused by pSS is not uncommon (50–52). Their clinical presentations can be and complex and variable, and treatment options should be carefully selected.

Myocardial damage in patients with pSS includes myocarditis, myocardial fibrosis, myocardial ischemia, etc., which are mainly caused by pSS related chronic inflammatory reactions. Myocardial fibrosis gradually appears when exposed to an inflammatory environment, such as increased TNFα, IL-1β, and IL-6 cytokine expression, as well as upregulated monocyte chemoattractant protein 1, IL-8, and biglycan expression, leading to overexpression of α-SMA, osteopontin, and lysy-l oxidase (53). Some scholars believe that vasculitis is caused by vascular wall injury followed by immune complexes formation secondary to immune-mediated antigen-antibody reactions. This process leads to vascular intimal thickening or even hyalinization, I and in severe cases, full-thickness vasculitis may occur, blocking the myocardial blood supply.

Patients with pSS often have abnormalities in their annular apparatus, such as valves, annulus, chordae tendineae, and papillary muscles, on echocardiography. Heart valve changes mainly manifested as valvular regurgitation. Abnormal valvular thickening in pSS patients mainly involve mitral, tricuspid and aortic valves. These thickening can be seen on the entire leaflets or along the margins of the leaflets, the development of which are thought to be dependent on the immunopathological features of pSS (54). Chronic inflammation associated with pSS immunoreactivity may contribute to exacerbated systemic sclerosis (SSc) in valvular tissue degeneration. In conclusion, for patients with confirmed pSS, attention should also be paid to their cardiac symptoms and signs in order to perform relevant examinations and decide for treatment

## Systemic sclerosis

SSc is clinically characterized by localized or diffuse thickening and fibrosis of the skin and systemic diseases affecting the heart, lungs, and digestive system. Extensive vascular disease, collagen proliferation, and fibrosis of the affected tissue are the pathological features of this disease. SSc often involves the heart, causing patients to develop cardiovascular disease while developing corresponding autoimmune symptoms. Anti-endothelial antibody-induced endothelial cell injury, ischemia/reperfusion injury, and immune-mediated cytotoxicity are the main causes of vascular injury, as well as impaired vascular repair mechanisms (55). Cardiac injury occurs in two forms: one is direct damage to the heart, including the production of large amounts of collagen fibers through the continuous activation of fibroblasts, impairment of microcirculatory function and immune system regulation. These can lead to ischemic damage of the myocardial tissue, fibrosis, small coronary artery spasm, valvular stenosis, and fibrosis or inflammatory damage to the pericardium and cardiac conduction system, the other is heart disease secondary to SSc damage of the lungs or kidneys (56). SSc can cause collagen proliferation and fibrosis in all parts of the heart, and then the corresponding clinical symptoms appear. Myocardium, coronary arteries, pericardium, cardiac conduction system and heart valves can also be affected.

Myocardial fibrosis is a typical presentation of cardiac involvement in SSc, it can manifest as patchy myocardial fibrosis, focal degeneration and necrosis (57). Myocardial fibrosis is often located under the endocardium, leading to a series of clinical symptoms such as myocardial ischemia, hypertrophy, and ventricular diastolic dysfunction. Bulkley reported that 50% of 52 SSc autopsy materials had myocardial lesions and multiple irregular patchy fibrotic lesions in the myocardium (58). Myocardial involvement is characterized by decreased myocardial systolic function and compliance. This may be the results of severe proliferation of collagen fibers in the myocardium. As recurrent Raynaud’s phenomenon in the myocardium leads to myocardial ischemia, healthy tissues are replaced by collagen fibers after degeneration and necrosis to form scars of different sizes.

## Conclusions

In recent years, cardiac damage in autoimmune diseases has gradually become the focus of attention of rheumatologists. Research on the pathogenesis, diagnosis and treatment of heart damage in autoimmune diseases is still in the exploratory stage. Immunosenescence is a process related to aging. T cells changes caused by immunosenescence have been shown to reduce normal immune responses as well as increase the risk for autoimmunity and inflammation. Various factors may contribute to immunosenescence related T cell changes, such as thymic degeneration, shortened telomere and epigenetic changes. It has also been reported that chronic viral infections may drive immunosenescence and contribute to the development of atherosclerosis (65).

Immunosenescence process is not due to overall functional decline of T cells, instead, it is due to the accumulation of T cells have undergone cellular senescence (66). Those senescent T cells have important roles in the pathophysiological processes of cardiovascular disease: they trigger inflammatory responses and cause cytotoxicity; both promote the progression of disease. At the same time, risk factors associated with cardiovascular diseases are also strongly related to senescent T cells. As we age, pro-inflammatory cytokines accumulate and cause increase in monocyte specific TLR signaling. This is shown to be linked with the development of chronic heart failure (67). It is not clear what the exact mechanism underlying autoimmune disease induced cardiac damage is. Recent studies have shed light on some cellular pathways: the role of adaptive immunity in cardiomyocyte injury, especially the roles of pro-inflammatory and anti-apoptotic phenotype of CD4 CD28 cytotoxic T cells (68). It has been reported that RA patients with extraarticular inflammation or atherosclerosis have prominent CD4+CD28-T cells (69). Chronic simulation of inflammatory factors in autoimmune disease can cause inflammatory damage of cardiomyocytes, which lead to cardiac damage. Cardiac dysfunction caused by inflammation have been reported in animal models as well (70). The importance of senescent immunity in autoimmune disease heart damage has not been officially proven and requires further research, which also provides another direction for our future research. The early clinical manifestations of heart injury in autoimmune diseases are relatively insidious and are not easy to attract the attention of physicians. When symptoms become apparent, the damage is often severe and irreversible; therefore, early diagnosis, timely prevention and treatment are essential to improve prognosis and reduce long-term morbidity and mortality. Therefore, the clinical manifestations and pathogenesis of cardiac damage in autoimmune diseases should be fully grasped, and cardiac damage in patients with autoimmune diseases should be treated with caution. After excluding their traditional risk factors for heart disease, the effects of the primary disease should be considered, and early examination, early detection and treatment should be carried out.

## Author contributions

Conceptualization: YZ and ZY; Methodology: S-YP and R-JC; Formal analysis: ZY and S-YP; Writing original draft preparation: H-MT, HZ, W-JG, S-YP, YZ; Writing and editing: S-YP, X-QW, R-JC. All authors contributed to the article and approved the submitted version.

## Funding

Seedling project of science and Technology Department of Sichuan Province (Project no. 2021JDRC0153) to S-YP.

## Conflict of interest

The authors declare that the research was conducted in the absence of any commercial or financial relationships that could be construed as a potential conflict of interest.

## Publisher’s note

All claims expressed in this article are solely those of the authors and do not necessarily represent those of their affiliated organizations, or those of the publisher, the editors and the reviewers. Any product that may be evaluated in this article, or claim that may be made by its manufacturer, is not guaranteed or endorsed by the publisher.

## References

[1] Damoiseaux J Andrade LE Fritzler MJ Shoenfeld Y . Autoantibodies 2015: From diagnostic biomarkers toward prediction, prognosis and prevention. Autoimmun Rev (2015) 14:555–63. doi: 10.1016/j.autrev.2015.01.017 25661979

[2] Jain D Halushka MK . Cardiac pathology of systemic lupus erythematosus. J Clin Pathol (2009) 62:584–92. doi: 10.1136/jcp.2009.064311 19561227

[3] Mavrogeni S Dimitroulas T Sfikakis PP Kitas GD . Heart involvement in rheumatoid arthritis: multimodality imaging and the emerging role of cardiac magnetic resonance. Semin Arthritis Rheumatism (2013) 43:314–24. doi: 10.1016/j.semarthrit.2013.05.001 23786873

[4] Castaneda S Gonzalez-Juanatey C Gonzalez-Gay MA . Inflammatory arthritis and heart disease. Curr Pharm Des (2018) 24:262–80. doi: 10.2174/1381612824666180123102632 29359662

[5] Gabriel SE . Cardiovascular morbidity and mortality in rheumatoid arthritis. Am J Med (2008) 121:S9–14. doi: 10.1016/j.amjmed.2008.06.011 PMC2858687 18926169

[6] Holmstrom M Koivuniemi R Korpi K Kaasalainen T Laine M Kuuliala A . Cardiac magnetic resonance imaging reveals frequent myocardial involvement and dysfunction in active rheumatoid arthritis. Clin Exp Rheumatol (2016) 34:416–23. 27050802

[7] Voskuyl AE . The heart and cardiovascular manifestations in rheumatoid arthritis. Rheumatol (Ox Eng) (2006) 45 Suppl 4:iv4–7. doi: 10.1093/rheumatology/kel313 16980723

[8] Nurmohamed MT . Cardiovascular risk in rheumatoid arthritis. Autoimmun Rev (2009) 8:663–7. doi: 10.1016/j.autrev.2009.02.015 19393192

[9] Myasoedova E Crowson CS Turesson C Gabriel SE Matteson EL . Incidence of extraarticular rheumatoid arthritis in Olmsted county, Minnesota, in 1995-2007 versus 1985-1994: A population-based study. J Rheumatol (2011) 38:983–9. doi: 10.3899/jrheum.101133 PMC3193155 21459933

[10] Gonzalez A Maradit Kremers H Crowson CS Ballman KV Roger VL Jacobsen SJ . Do cardiovascular risk factors confer the same risk for cardiovascular outcomes in rheumatoid arthritis patients as in non-rheumatoid arthritis patients? Ann Rheum Dis (2008) 67:64–9. doi: 10.1136/ard.2006.059980 17517756

[11] Di Franco M Paradiso M Mammarella A Paoletti V Labbadia G Coppotelli L . Diastolic function abnormalities in rheumatoid arthritis. evaluation by echo Doppler transmitral flow and pulmonary venous flow: relation with duration of disease. Ann Rheum Dis (2000) 59:227–9. doi: 10.1136/ard.59.3.227 PMC1753085 10700433

[12] Roubille C Richer V Starnino T McCourt C McFarlane A Fleming P . The effects of tumour necrosis factor inhibitors, methotrexate, non-steroidal anti-inflammatory drugs and corticosteroids on cardiovascular events in rheumatoid arthritis, psoriasis and psoriatic arthritis: a systematic review and meta-analysis. Ann Rheum Dis (2015) 74:480–9. doi: 10.1136/annrheumdis-2014-206624 PMC4345910 25561362

[13] Gasparyan AY Cocco G Pandolfi S . Cardiac complications in rheumatoid arthritis in the absence of occlusive coronary pathology. Rheumatol Int (2012) 32:461–4. doi: 10.1007/s00296-009-1318-4 20091033

[14] Koivuniemi R Paimela L Suomalainen R Leirisalo-Repo M . Cardiovascular diseases in patients with rheumatoid arthritis. Scand J Rheumatol (2013) 42:131–5. doi: 10.3109/03009742.2012.723747 23244227

[15] Turiel M Sitia S Tomasoni L Cicala S Atzeni F Gianturco L . [Cardiac involvement in rheumatoid arthritis]. Reumatismo (2009) 61:244–53. doi: 10.4081/reumatismo.2009.244 20143001

[16] Thadani U Iveson JM Wright V . Cardiac tamponade, constrictive pericarditis and pericardial resection in rheumatoid arthritis. Medicine (1975) 54:261–70. doi: 10.1097/00005792-197505000-00006 1143088

[17] Jordan AD Khan ME Hoey ET Rassl D Nashef SA . A clinico-pathological conference on constrictive pericarditis secondary to rheumatoid arthritis: a case report with expert commentary and review of the literature. Heart Lung Circ (2011) 20:24–9. doi: 10.1016/j.hlc.2010.08.014 20851679

[18] Rawla P . Cardiac and vascular complications in rheumatoid arthritis. Reumatologia (2019) 57:27–36. doi: 10.5114/reum.2019.83236 30858628 PMC6409824

[19] Lazzerini PE Capecchi PL Laghi-Pasini F Boutjdir M . Autoimmune channelopathies as a novel mechanism in cardiac arrhythmias. Nat Rev Cardiol (2017) 14:521–35. doi: 10.1038/nrcardio.2017.61 28470179

[20] Lazzerini PE Capecchi PL Laghi-Pasini F . Systemic inflammation and arrhythmic risk: lessons from rheumatoid arthritis. Eur Heart J (2017) 38:1717–27. doi: 10.1093/eurheartj/ehw208 27252448

[21] Lazzerini PE Capecchi PL El-Sherif N Laghi-Pasini F Boutjdir M . Emerging arrhythmic risk of autoimmune and inflammatory cardiac channelopathies. J Am Heart Assoc (2018) 7:e010595. doi: 10.1161/JAHA.118.010595 30571503 PMC6404431

[22] Turk SA Heslinga SC Dekker J Britsemmer L van der Lugt V Lems WF . The relationship between cardiac conduction times, cardiovascular risk factors, and inflammation in patients with early arthritis. J Rheumatol (2017) 44:580–6. doi: 10.3899/jrheum.161184 28365582

[23] Mullins PA Grace AA Stewart SC Shapiro LM . Rheumatoid heart disease presenting as acute mitral regurgitation. Am Heart J (1991) 122:242–5. doi: 10.1016/0002-8703(91)90789-K 2063749

[24] Hansson GK . Inflammation, atherosclerosis, and coronary artery disease. New Engl J Med (2005) 352:1685–95. doi: 10.1056/NEJMra043430 15843671

[25] Gupta S Jesrani G Gaba S Gupta M Kumar S . Constrictive pericarditis as an initial manifestation of systemic lupus erythematosus. Cureus (2020) 12:e11256. doi: 10.7759/cureus.11256 33269173 PMC7707119

[26] Shazzad MN Islam MN Ara R Ahmed CM Fatema N Azad AK . Echocardiographic assessment of cardiac involvement in systemic lupus erythematosus patients. Mymensingh Med J (2013) 22:736–41. 24292305

[27] Jia E Geng H Liu Q Xiao Y Zhang Y Xie J . Cardiac manifestations of han Chinese patients with systemic lupus erythematosus: a retrospective study. Ir J Med Sci (2019) 188:801–6. doi: 10.1007/s11845-018-1934-7 30460452

[28] Lindop R Arentz G Thurgood LA Reed JH Jackson MW Gordon TP . Pathogenicity and proteomic signatures of autoantibodies to ro and la. Immunol Cell Biol (2012) 90:304–9. doi: 10.1038/icb.2011.108 22249199

[29] Quismorio FP Jr . Immune complexes in the pericardial fluid in systemic lupus erythematosus. Arch Intern Med (1980) 140:112–4. doi: 10.1001/archinte.1980.00330130114028 6965447

[30] Panchal L Divate S Vaideeswar P Pandit SP . Cardiovascular involvement in systemic lupus erythematosus: an autopsy study of 27 patients in India. J Postgrad Med (2006) 52:5–10; discussion 10. 16534157

[31] Cooper LT Jr . Myocarditis. N Engl J Med (2009) 360:1526–38. doi: 10.1056/NEJMra0800028 PMC5814110 19357408

[32] Tariq S Garg A Gass A Aronow WS . Myocarditis due to systemic lupus erythematosus associated with cardiogenic shock. Arch Med Sci (2018) 14:460–2. doi: 10.5114/aoms.2017.68692 PMC5868675 29593821

[33] Maceira AM Prasad SK Khan M Pennell DJ . Normalized left ventricular systolic and diastolic function by steady state free precession cardiovascular magnetic resonance. J Cardiovasc Magn Reson (2006) 8:417–26. doi: 10.1080/10976640600572889 16755827

[34] Cervera R Font J Pare C Azqueta M Perez-Villa F Lopez-Soto A . Cardiac disease in systemic lupus erythematosus: prospective study of 70 patients. Ann Rheum Dis (1992) 51:156–9. doi: 10.1136/ard.51.2.156 PMC1005649 1550395

[35] Chang JC Xiao R Mercer-Rosa L Knight AM Weiss PF . Child-onset systemic lupus erythematosus is associated with a higher incidence of myopericardial manifestations compared to adult-onset disease. Lupus (2018) 27:2146–54. doi: 10.1177/0961203318804889 PMC6207474 30318995

[36] Finkelstein Y Adler Y Harel L Nussinovitch M Youinou P . Anti-ro (SSA) and anti-la (SSB) antibodies and complete congenital heart block. Ann Med Interne (Paris) (1997) 148:205–8. 9255327

[37] Shapiro RF Gamble CN Wiesner KB Castles JJ Wolf AW Hurley EJ . Immunopathogenesis of libman-sacks endocarditis. assessment by light and immunofluorescent microscopy in two patients. Ann Rheum Dis (1977) 36:508–16. doi: 10.1136/ard.36.6.508 PMC1000155 339850

[38] Roldan CA Shively BK Crawford MH . An echocardiographic study of valvular heart disease associated with systemic lupus erythematosus. New Engl J Med (1996) 335:1424–30. doi: 10.1056/NEJM199611073351903 8875919

[39] Lazzerini PE Capecchi PL Guideri F Bellisai F Selvi E Acampa M . Comparison of frequency of complex ventricular arrhythmias in patients with positive versus negative anti-Ro/SSA and connective tissue disease. Am J Cardiol (2007) 100:1029–34. doi: 10.1016/j.amjcard.2007.04.048 17826392

[40] Logar D Kveder T Rozman B Dobovisek J . Possible association between anti-ro antibodies and myocarditis or cardiac conduction defects in adults with systemic lupus erythematosus. Ann Rheum Dis (1990) 49:627–9. doi: 10.1136/ard.49.8.627 PMC1004179 2396870

[41] Toh N Kanzaki H Nakatani S Ohara T Kim J Kusano KF . Left atrial volume combined with atrial pump function identifies hypertensive patients with a history of paroxysmal atrial fibrillation. Hypertension (2010) 55:1150–6. doi: 10.1161/HYPERTENSIONAHA.109.137760 20368506

[42] Davis JM 3rd Roger VL Crowson CS Kremers HM Therneau TM Gabriel SE . The presentation and outcome of heart failure in patients with rheumatoid arthritis differs from that in the general population. Arthritis Rheum (2008) 58:2603–11. doi: 10.1002/art.23798 PMC2631416 18759286

[43] Mavrogeni S Koutsogeorgopoulou L Dimitroulas T Markousis-Mavrogenis G Kolovou G . Complementary role of cardiovascular imaging and laboratory indices in early detection of cardiovascular disease in systemic lupus erythematosus. Lupus (2017) 26:227–36. doi: 10.1177/0961203316671810 27687024

[44] Parkes JE Day PJ Chinoy H Lamb JA . The role of microRNAs in the idiopathic inflammatory myopathies. Curr Opin Rheumatol (2015) 27:608–15. doi: 10.1097/BOR.0000000000000225 26398013

[45] Hughes M Lilleker JB Herrick AL Chinoy H . Cardiac troponin testing in idiopathic inflammatory myopathies and systemic sclerosis-spectrum disorders: biomarkers to distinguish between primary cardiac involvement and low-grade skeletal muscle disease activity. Ann Rheum Dis (2015) 74:795–8. doi: 10.1136/annrheumdis-2014-206812 PMC4589894 25732174

[46] Fairley JL Wicks I Peters S Day J . Defining cardiac involvement in idiopathic inflammatory myopathies: a systematic review. Rheumatol (Oxford England) (2021) 61:103–20. doi: 10.1093/rheumatology/keab573 34273157

[47] Dhir T Jiang N . Misleading elevation of troponin T caused by polymyositis. Int J BioMed Sci (2013) 9:107–11. PMC3708267 23847461

[48] Lundberg IE . The heart in dermatomyositis and polymyositis. Rheumatol (Oxford England) (2006) 45 Suppl 4:iv18–21. doi: 10.1093/rheumatology/kel311 16980718

[49] Danieli MG Gelardi C Guerra F Cardinaletti P Pedini V Gabrielli A . Cardiac involvement in polymyositis and dermatomyositis. Autoimmun Rev (2016) 15:462–5. doi: 10.1016/j.autrev.2016.01.015 26826433

[50] Casian M Jurcut C Dima A Mihai A Stanciu S Jurcut R . Cardiovascular disease in primary sjogren's syndrome: Raising clinicians' awareness. Front Immunol (2022) 13:865373. doi: 10.3389/fimmu.2022.865373 35757738 PMC9219550

[51] Yong WC Sanguankeo A Upala S . Association between primary sjogren's syndrome, arterial stiffness, and subclinical atherosclerosis: a systematic review and meta-analysis. Clin Rheumatol (2019) 38:447–55. doi: 10.1007/s10067-018-4265-1 30178172

[52] Tsai YD Chien WC Tsai SH Chung CH Chu SJ Chen SJ . Increased risk of aortic aneurysm and dissection in patients with sjogren's syndrome: a nationwide population-based cohort study in Taiwan. BMJ Open (2018) 8:e022326. doi: 10.1136/bmjopen-2018-022326 PMC6157519 30244213

[53] Suthahar N Meijers WC Sillje HHW de Boer RA . From inflammation to fibrosis-molecular and cellular mechanisms of myocardial tissue remodelling and perspectives on differential treatment opportunities. Curr Heart Fail Rep (2017) 14:235–50. doi: 10.1007/s11897-017-0343-y PMC5527069 28707261

[54] Espinola Zavaleta N Morales Blanhir J de Witt Garcia M Romero Cardenas A Vargas Barron J . [Echocardiographic findings in primary sjogren syndrome]. Arch Inst Cardiol Mex (1997) 67:114–25. doi: 10.3978/j.issn.2305-5839.2014.12.12 9412422

[55] Cannarile F Valentini V Mirabelli G Alunno A Terenzi R Luccioli F . Cardiovascular disease in systemic sclerosis. Ann Transl Med (2015) 3:8. doi: 10.3978/j.issn.2305-5839.2014.12.12 25705640 PMC4293487

[56] Ferri C Valentini G Cozzi F Sebastiani M Michelassi C La Montagna G . Systemic sclerosis: demographic, clinical, and serologic features and survival in 1,012 Italian patients. Medicine (2002) 81:139–53. doi: 10.1097/00005792-200203000-00004 11889413

[57] Kurmann RD El-Am EA Radwan YA Sandhu AS Crowson CS Matteson EL . Increased risk of valvular heart disease in systemic sclerosis: An underrecognized cardiac complication. J Rheumatol (2021) 48:1047–52. doi: 10.3899/jrheum.201005 PMC8254733 33452164

[58] Bulkley BH Ridolfi RL Salyer WR Hutchins GM . Myocardial lesions of progressive systemic sclerosis. a cause of cardiac dysfunction. Circulation (1976) 53:483–90. doi: 10.1161/01.CIR.53.3.483 1248080

[59] Smolen JS Aletaha D McInnes IB . Rheumatoid arthritis. Lancet (London England) (2016) 388:2023–38. doi: 10.1016/S0140-6736(16)30173-8 27156434

[60] Mantel A Holmqvist M Andersson DC Lund LH Askling J . Association between rheumatoid arthritis and risk of ischemic and nonischemic heart failure. J Am Coll Cardiol (2017) 69:1275–85. doi: 10.1016/j.jacc.2016.12.033 28279294

[61] Kessler J Totoson P Devaux S Moretto J Wendling D Demougeot C . Animal models to study pathogenesis and treatments of cardiac disorders in rheumatoid arthritis: Advances and challenges for clinical translation. Pharmacol Res (2021) 170:105494. doi: 10.1016/j.phrs.2021.105494 34139344

[62] Dai H Wang X Yin S Zhang Y Han Y Yang N . Atrial fibrillation promotion in a rat model of rheumatoid arthritis. J Am Heart Assoc (2017) 6 :e007320. doi: 10.1161/JAHA.117.007320 29269354 PMC5779041

[63] Avina-Zubieta JA To F Vostretsova K De Vera M Sayre EC Esdaile JM . Risk of myocardial infarction and stroke in newly diagnosed systemic lupus erythematosus: A general population-based study. Arthritis Care Res (2017) 69:849–56. doi: 10.1002/acr.23018 28129475

[64] Riviere E Cohen Aubart F Maisonobe T Maurier F Richez C Gombert B . Clinicopathological features of multiple mononeuropathy associated with systemic lupus erythematosus: a multicenter study. J Neurol (2017) 264:1218–26. doi: 10.1007/s00415-017-8519-7 28536920

[65] Ghamar Talepoor A Doroudchi M . Immunosenescence in atherosclerosis: A role for chronic viral infections. Front Immunol (2022) 13:945016. doi: 10.3389/fimmu.2022.945016 36059478 PMC9428721

[66] Shirakawa K Sano M . T Cell immunosenescence in aging, obesity, and cardiovascular disease. Cells (2021) 10 :24357. doi: 10.3390/cells10092435 PMC8464832 34572084

[67] Wang Y Dong C Han Y Gu Z Sun C . Immunosenescence, aging and successful aging. Front Immunol (2022) 13:942796. doi: 10.3389/fimmu.2022.942796 35983061 PMC9379926

[68] Van Laecke S Malfait T Schepers E Van Biesen W . Cardiovascular disease after transplantation: an emerging role of the immune system. Transpl Int (2018) 31:689–99. doi: 10.1111/tri.13160 29611220

[69] Petersen LE Grassi-Oliveira R Siara T dos Santos SG Ilha M de Nardi T . Premature immunosenescence is associated with memory dysfunction in rheumatoid arthritis. Neuroimmunomodulation (2015) 22:130–7. doi: 10.1159/000358437 24751698

[70] Zhou Z Miao Z Luo A Zhu D Lu Y Li P . Identifying a marked inflammation mediated cardiac dysfunction during the development of arthritis in collagen-induced arthritis mice. Clin Exp Rheumatol (2020) 38:203–11. doi: 10.55563/clinexprheumatol/6kxs1o 31140393

